# Assessing the Quality of the World Health Organization’s Skin NTDs App as a Training Tool in Ghana and Kenya: Protocol for a Cross-sectional Study

**DOI:** 10.2196/39393

**Published:** 2022-12-08

**Authors:** Asmae Frej, Mireia Cano, José A Ruiz-Postigo, Paul Macharia, Richard Odame Phillips, Yaw Ampem Amoako, Carme Carrion

**Affiliations:** 1 School of Health Sciences Universitat de Girona Girona Spain; 2 eHealth Lab Research Group eHealth Center & School of Health Sciences Universitat Oberta de Catalunya Barcelona Spain; 3 Prevention, Treatment and Care Unit Department of Control of Neglected Tropical Diseases World Health Organization Geneve Switzerland; 4 Consulting in Health Informatics Nairobi Kenya; 5 Kumasi Center for Collaborative Research in Tropical Medicine Kumasi Ghana

**Keywords:** Skin NTDs App, mHealth, mobile health, neglected tropical diseases, skin neglected tropical diseases, low- and middle-income countries

## Abstract

**Background:**

Neglected tropical diseases (NTDs) affect over 1.5 billion people worldwide, the majority of them belonging to impoverished populations in low- and middle-income countries (LMICs). Skin NTDs are a subgroup of NTDs that manifest primarily as skin lesions. The diagnosis and treatment of skin NTDs entail considerable resources, including trained personnel and financial backing. Many interventions are being launched and evaluated, particularly mobile health (mHealth) interventions, such as Skin NTDs App, a training and decision support tool offered by the World Health Organization (WHO) for frontline health workers (FHWs). As most digital health guidelines prioritize the thorough evaluation of mHealth interventions, it is essential to conduct a rigorous and validated assessment of Skin NTDs App.

**Objective:**

We aim to assess the quality of version 3 of Skin NTDs App, developed for the WHO by Universal Doctor and Netherlands Leprosy Relief as a training and decision support tool for FHWs.

**Methods:**

A cross-sectional study will be conducted in 2 LMICs: Ghana and Kenya. We will use snowball sampling recruitment to select 48 participants from the target population of all FHWs dealing with skin NTDs. The sample group of FHWs will be asked to download and use Skin NTDs App for at least 5 days before answering a web-based survey containing demographic variables and the user Mobile App Rating Scale (uMARS) questionnaire. A semistructured interview will then be conducted. Quantitative and qualitative data will be analyzed using SPSS (version 25; SPSS Inc), with statistical significance for all tests set at a 95% CI and *P*≤.05 considered significant. Data derived from the semistructured interviews will be clustered in themes and coded to enable analysis of various dimensions using ATLAS.ti.

**Results:**

The estimated completion date of the study is in the third quarter of 2022. The results are expected to show that Skin NTDs App version 3 has a good reported user experience, as assessed using the uMARS scale. No differences are expected to be found, except for those related to experience in dermatology and the use of mobile technology that could influence the final score. Semistructured interviews are expected to complete the results obtained on the uMARS scale. Moreover, they will be the previous step before assessing other aspects of the app, such as its efficiency and how it should be disseminated or implemented.

**Conclusions:**

This study is the first step in a qualitative and quantitative assessment of Skin NTDs App as a training and support tool for FHWs diagnosing and managing skin NTDs. Our results will serve to improve future versions of the App.

**International Registered Report Identifier (IRRID):**

PRR1-10.2196/39393

## Introduction

Neglected tropical diseases (NTDs) are a group of 20 diseases and conditions identified by the World Health Organization (WHO), which affect over 1.5 billion people. Most of afflicted individuals are women and children living in impoverished populations in tropical and subtropical regions [1]. If not detected or treated, some of these diseases are fatal or can become chronic and irreversible, not only causing lifelong disabilities but also stigma and social exclusion, thereby perpetuating a cycle of poverty with a direct impact on development and economic productivity in low- and middle-income countries (LMICs) [2]. The combination of these factors renders the control of NTDs both necessary and complex.

NTDs had not received global prioritization until 2005, when the WHO established a new strategy to target the group as a whole [1]. Now, NTDs are classified in 2 groups: those that are potentially preventable through large-scale chemotherapy interventions and those that can only be addressed through individual case management [3].

Skin NTDs are a subgroup of NTDs that manifest primarily as lesions on the skin and can be detected through visual screening [4,5]. This group consists of Buruli ulcer, cutaneous leishmaniasis, deep fungal infections, post-kala-azar dermal leishmaniasis, leprosy, lymphatic filariasis, mycetoma, onchocerciasis, scabies and other ectoparasites, and yaws. As most of these conditions do not benefit from large-scale preventive drug administration, they rely on early diagnosis and treatment, which, in turn, relies on considerable resources, including trained personnel and financial backing [4].

Frontline health workers (FHWs) have been identified as vital to the impact of any campaign intended to tackle NTDs [6]. The term FHWs refers to any health worker who directly provides service to a community. As they often have insufficient specialized medical knowledge, data collection procedures, and contact with peers, FHWs need to be equipped with knowledge and tools that will facilitate their role in diagnosing, treating, and referring patients to another level of the health system.

New methods to improve the clinical management and epidemiological surveillance of many diseases, including infectious and skin diseases, have recently been developed [7]. The WHO has recognized that mobile health (mHealth) can be a significant component in delivering global health care and can provide considerable support for FHWs [8]. However, the most recent systematic review of mHealth strategies for dealing with skin NTDs concluded that work is needed to homogenize interventions and thereby reduce methodological limitations [9].

To support FHWs with new technologies, the WHO’s Department of Control of NTDs has developed Skin NTDs App [10], a mobile version of the training guide they published in 2018 [11]. This App helps FHWs diagnose and manage skin NTDs by using an algorithm based on identifying signs and symptoms and provides extra information about these diseases. The recently released version 3, a combination of WHO’s Skin NTDs and SkinApp*,* developed by the nongovernmental organization (NGO) Netherlands Leprosy Relief, has several new features. As yet, the new app is not publicly available, and no evaluations have been conducted. Moreover, studies have shown that relying on the system of “star”-rating iOS and Android devices is insufficient within the exponentially expanding market of medical apps [12].

Most digital health technology frameworks include evaluation of mHealth interventions as a crucial step in ensuring quality and enabling end users to not rely solely on popularity [13-15]. At this point, it is important to emphasize that quality refers to not only clinical effectiveness but also other important domains such as, among others, usability, interoperability, technical security, and data protection [13]. The user version of the Mobile App Rating Scale (uMARS) [12] has been found to be a simple and reliable tool for classifying and rating mobile health apps based on objective and subjective quality domains [14,15].

As the developers of Skin NTDs App have ambitious plans and expectations, it is essential to conduct a rigorous and validated assessment of this app. This paper describes the protocol for a cross-sectional study aimed to assess the engagement, functionality, aesthetics, and quality information for the real end user in their actual context of version 3 of Skin NTDs App in accordance with a validated tool. In addition, a secondary goal of this study will be to determine whether any of the demographic information collected has an impact on the final uMARS score, since the developers of Skin NTDs App do not intend to customize the app when implementing it in any setting.

## Methods

### Study Design

Between April 2022 and May 2022, we will conduct a cross-sectional study among 98 FHWs based in 2 LMICs: Ghana and Kenya.

### Ethics Approval

This study will be conducted in accordance with the ethical principles established by the World Medical Association in the *Declaration of Helsinki–Ethical Principles for Medical Research Involving Human Subjects* [16]. This protocol has been approved by the ethics committee of Universitat Oberta de Catalunya.

Additionally, to ensure that the protocol also meets the ethical requirements in the countries where the study will be conducted, it has been already submitted in Ghana to the Ethical Committee of Kwame Nkrumah University of Science and Technology, Kumasi. The authors have also initiated the process to submit the protocol in Kenya to the Ethical Committee of Coast General Teaching and Referral Hospital, Mombasa.

### Participants and Eligibility

All FHWs who deal with skin NTDs in both selected countries on a daily basis, are responsible for the diagnosis and management of skin NTDs, who own a smartphone device (Apple or Android), and have downloaded Skin NTDs App and used it on at least 5 different days for at least 10 minutes each day are considered eligible. Moreover, participants will need to have access to email or WhatsApp to provide informed consent.

Exclusion criteria are a low comprehension of the English language and refusal to sign the informed consent form.

The target population is the same for both parts of the study. Participants will be asked to read the information sheet (for more detail, see Multimedia Appendix 1) before signing the informed consent form.

### Sample Recruitment

The difficulty of having direct contact with FHWs in these 2 countries necessitates nonprobabilistic snowball sampling, a recruitment technique in which selected participants are asked to identify and contact other potential subjects among their acquaintances to participate in the study. Despite the fact that this method is nonrandomized, it seems to be the most effective way to identify a difficult-to-reach audience, when participants are contacted digitally, and researchers are located in another continent.

**Figure 1:**
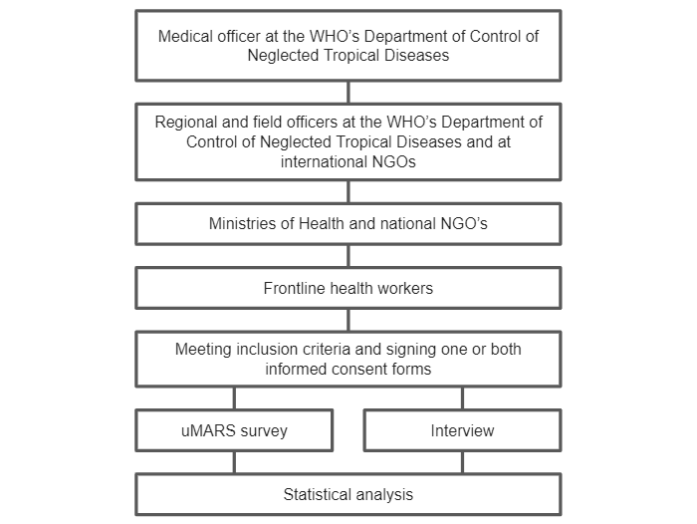
Diagrammatic representation of snowball sampling recruitment. uMARS: user Mobile Application Rating Scale; WHO: World Health Organization.

The chain of contacts will originate from a medical officer in WHO’s Department of Control of NTDs, who will contact colleagues who work on skin NTDs in the WHO regional and country offices and international NGOs by mail. The country offices will be asked to invite FHWs to participate in the study through Ministries of Health or national NGOs. All FHWs who meet the inclusion criteria and sign the informed consent form will be sent the survey and, if applicable, scheduled for interview (Figure 1).

### Sample Size Calculation

Sample size was calculated on the basis of the 6 stages of the intervention maturity life cycle described by the WHO in *Monitoring and Evaluating Digital Health Interventions* [17], a schematic and practical guide to know which have to be the goals in each stage, how many participants are required, and which are the measurement targets.

Skin NTDs App is currently in the prototype phase, which corresponds to stages 1 and 2 of the 6 stages of the intervention maturity life cycle, according to WHO guidelines. As a result, the WHO advises testing it with a sample size of between 10 and 100 users.

Taking into account that 100 is the maximum recommended number of participants and the web-based modality of this study, we assume a dropout rate of 50%. These calculations leave a final sample size of 50 participants.

### Outcomes

#### Demographic Variables

Participants will be asked to complete an anonymous survey of various demographic data, which will include the following: age, gender (“Male,” “Female,” “nonbinary,” and “transgender”), country of residence (“Ghana” and “Kenya”), type of FHWs (3 specified categories will be provided as these are most common: “Medical Doctor,” “Nurse,” “Community Health Worker,” and “Other” under which participants will be asked to add their own category), frequency of dealing with skin NTDs [“Rarely (<1 case/month),” “Occasionally (1-3 cases/month),” “Frequently (4-6 cases/month),” and “Usually (>6 cases/month)”], experience and training in dermatology (“Trained and experienced,” “Not trained but with experience with dermatology patients,” and “Neither trained nor experienced”), work environment (“rural” and “urban”), working institution (“Public healthcare setting,” “Private healthcare setting,” and “NGO”), knowledge of mobile technology (“High—I’m able to use mobile technologies without having many issues,” “Medium—I’m able to use mobile technologies but sometimes I need some help,” and “Low—I’m not able to use mobile technologies without supervision and help”), and languages (open answer for participants to enter the languages they can speak).

#### uMARS Questionnaire

The uMARS is a simple, objective, and reliable tool developed for users by Stoyanov et al [12] to classify and assess the quality of health-related mobile apps. The tool assesses 20 items clustered into 4 objective subscales (engagement, functionality, aesthetics, and information quality) and 1 subjective subscale. Participants rate each item using a Likert scale from 1 to 5 (1=“Inadequate,” 2=“Poor,” 3=“Acceptable,” 4=“Good,” and 5=“Excellent”). There is also the option “not applicable” if an item cannot be assessed. There is an extra category entitled *App-Specific* with descriptive purposes only that contains 5 more items and can be adjusted and adapted by researchers. This category evaluates the perceived impact of the app on users’ knowledge, attitudes, intentions to change, and the likelihood of actual change. We will add 9 questions to complete this domain, relating to app discovery, time using the app, frequency of use, notification updates, usefulness, translation features, patient record features, and desktop version.

The uMARS score is calculated separately based on the original recommendation from the authors of this scale. Hence, 2 scores will be obtained: app quality mean score and app subjective quality score. This division is made to strengthen the objectivity of the uMARS as a measure of app quality. All those questions rated as “not applicable” will be excluded from the mean scores.

#### Semistructured Interviews

Semistructured interviews are a validated qualitative method for exploring the perspectives, perceptions, and opinions of participants. Semistructured interviews combine prepared questions with others that arise during the interview [18].

In this study, interviewers will ask 8 questions in the same order and using the same words to each participant—a standardization that will facilitate comparability [19]. Questions will be open-ended, neutral, clear, and in familiar language. The structure of the interviews will be as follows: a brief presentation of the interviewer and the study, a general question related to the overall use of Skin NTDs App, 8 core questions directly relating to information needed for app assessment, and unplanned questions that arise during the interview based on the participants’ answers.

It is estimated that semistructured interviews will be conducted in a minimum of the 10% of the final sample size or until information saturation is reached.

### Data Collection and Study Procedure

Once a participant has signed the initial consent form, an email will be sent with the first part of the survey and details of the study procedure. This information will include the following: links to download the app, a recommended time of app usage before answering the survey, and information about the interviews.

Participants will receive an email over the following days with a Google Forms link through which they can enter their demographic data and answers to the uMARS questionnaire. In the last section of the survey, participants will need to indicate whether they would like to participate in the next phase of the study—the semistructured interviews.

A separate email or WhatsApp message will be sent to those who wish to participate in the interviews. Participants will receive an information sheet and a second consent form, which must be signed and returned before the participant can be interviewed.

Once informed consent has been obtained, participants and researchers will schedule a semistructured interview at a mutually convenient time. Interviews will be carried out via video calls using Google Meets; therefore, participants will receive an email or WhatsApp message with a link specifying a date and time.

Once the interviewer has reminded the interviewee why audio recording is necessary, audio recording will start and continue throughout the interview. The questions will be asked as previously described (for more detail, see Multimedia Appendix 2). Interviews will last between 25 and 40 minutes, during which time the interviewer will take notes and repeat questions or words as required to fully understand the interviewee’s meaning.

Researchers will use Otter.ai software to transcribe all interviews for analysis. During the data collection phase of the study, researchers will send as many reminders to participants as they deem necessary.

### Statistical Analysis

Quantitative data will be collected in Excel (Microsoft Inc) to be analyzed using SPSS for Windows (version 25; SPSS Inc). Statistical significance for all tests will be set at a *P* value of ≤.05. To describe the quantitative information received from the uMARS scores, a descriptive analysis will be carried out. Measures of frequency, central tendency, and dispersion will be used to describe this data, which will be shown in tables. Depending on sample size, a Shapiro-Wilk or Kolmogorov-Smirnov test will be used to assess the normality of data (*P*≤.05). In the bivariate analysis, a chi-square test will be used to compare categorical variables. Depending on whether the distribution is normal, quantitative data will be compared using a Student *t* or Mann-Whitney *U* test. A multivariate analysis will be performed using a logistic regression analysis to add the covariates that could skew the main association under analysis. Finally, a cluster analysis will be performed to identify similar groups based on the observed values of several variables. A 95% CI will be assumed and *P*≤.05 will be considered to indicate a significant difference.

Selected quotes will be returned to participants for approval. Qualitative data derived from the semistructured interviews will be analyzed using ATLAS.ti (Scientific Software Development GmbH). We will identify attributes, cluster them into different themes, and then code these themes to analyze the various dimensions explored during the interviews.

## Results

The results of this work are expected during the third quarter of 2022. First, Skin NTDs App version 3 is expected to obtain a result of >3 out of 5 points on the uMARS. In addition, it is expected that the results will not show any difference by sex, type of work environment or working institution, or country. However, it is suspected that those variables related to experience in dermatology or the use of mobile technology may be determining factors when evaluating the user experience with the App. On the other hand, the perceptions collected from the semistructured interviews are expected to be the element to understand the reason for the results obtained on the uMARS.

Moreover, they will be the previous step before assessing other aspects of the App, such as the efficiency, how it should be disseminated or implemented, etc.

## Discussion

### Principal Findings

Skin NTDs comprise a subgroup of 13 NTDs that present primarily as lesions on the skin and can be detected through visual screening. They rely on early diagnosis and treatment, which implies that the resources consumed are significant. However, if not detected or treated, they can potentially turn chronic and irreversible, thus favoring a cycle of deteriorating health and socioeconomic status, which does nothing to help the overall development of LMICs.

Any solution that helps improve their current management should be considered and studied in detail. Among them, the WHO has identified the use of mHealth as a critical component in tackling the complexities underlying skin NTDs.

In this line of work, Skin NTDs App is an initiative by the WHO’s Department of Control of Neglected Tropical Diseases as a training and decision support tool to assist FHWs who diagnose and manage skin NTDs in their daily practice. Through the uMARS questionnaire and semistructured interviews, this novel cross-sectional study will investigate how FHWs quantitatively and qualitatively rate the quality of Skin NTDs App version 3.

Considering that the app is still under development and has not yet been implemented in countries where skin NTDs are prevalent, this study represents a chance to include the feedback of the real end users in upcoming versions. Moreover, this action is likely to influence the subsequent impact of any implementation campaigns that may be undertaken.

### Limitations

There are various limitations associated with this study.

The first limitation that arises and may seem relevant is the decision behind choosing Ghana and Kenya to perform this study. This was based on 3 factors: (1) Skin NTDs App can only be downloaded in English; hence, the choices are limited to countries where English is an official language; (2) these 2 countries have a long history of web-based disease tracking and are endemic for at least 8 skin NTDs each; and (3) to ensure an adequate sampling, countries were chosen on the basis of the accessibility to key officials and program managers and their chances to further contact enough respondents.

Regarding the methodology used, snowball sampling is a nonprobabilistic and nonrandomized method; hence, the sample may not be representative of the general population. However, because the study will be conducted entirely on the internet and the participants and authors are in different places, this is the only way we can connect with FHWs—a highly specialized group that can be challenging to reach in LMICs if contacted remotely.

On the other hand, it is also important to point out some limitations of the uMARS. First, this tool has only been validated among young people using 2 specific apps related to other health areas. However, its high reliability and the inclusion of both objective and subjective domains make it the only questionnaire available that is validated and simple for users to assess health apps.

Another requirement is that FHWs will have to use the app for at least 5 days as opposed to the 10 minutes originally required by the original uMARS publication. Since there will not be any direct control over that, this statement cannot be guaranteed. To determine whether they have fulfilled it or not, a question has been added to the survey.

Finally, it is important to highlight that this study does not claim to assess the clinical effectiveness of Skin NTDs App. This would require a randomized clinical trial and exceed the scope of this study. Although this is version 3, there are still issues to address, including whether information in the app is updated in response to the most recent research.

### Conclusions

In conclusion, the findings of this study will be used to enhance upcoming releases of Skin NTDs App by examining FHWs' perspectives. In the future, this tool might be made available to LMICs in an effort to improve the management of skin NTDs. Thus, to develop an app that has a larger chance of being used widely around the world, it is crucial to evaluate the true impact of Skin NTDs App on the real end users—that is, FHWs—and include their thoughts in this initial phase.

## Appendix

Multimedia Appendix 1 Information sheet.

Multimedia Appendix 2 Semi-structured interview.

## Abbreviations

FHW: frontline health worker
LMIC: low- and middle-income country
mHealth: mobile health
NGO: nongovernmental organization
NTD: neglected tropical disease
uMARS: user Mobile Application Rating Scale
WHO: World Health Organization

## Data Availability

All data sets generated during the study will be deposited in available repositories or added to the main manuscript when published. Data derived from the uMARS survey will be published in a data sheet file. On the other hand, data from the semistructured interviews will be shared in a document format only with the most relevant sentences or answers from participants. All data considered confidential will not be shared.
