# Supplementary material for: Assessing the Quality of the World Health Organization’s Skin NTDs App as a Training Tool in Ghana and Kenya: Protocol for a Cross-sectional Study
Source: JMIR Res Protoc. 2022 Dec 8;11(12):e39393. doi: 10.2196/39393 (PMC9782345; doi:10.2196/39393)
Supplement: Multimedia Appendix 1 [file resprot_v11i12e39393_app1.docx]

**MULTIMEDIA APPENDIX 1. Information sheet**

INFORMATION SHEET FOR THE INTERVIEWEE

Assessing the quality of the WHO Skin NTDs App as a training tool in four low and middle income countries: Protocol for a cross-sectional study.

*1. General information*

You are being invited to participate in the second part of our pilot test, where you will be asked several questions about your experience with the Skin NTDs app, in the form of a semi-structured interview.

The study is being conducted by Mireia Cano, research assistant from the Universitat Oberta de Catalonia (UOC), in collaboration with Dra. Carme Carrión, principal investigator of eHealth lab Research Group of the UOC and Dr. José Postigo, medical officer of the department of Control of Neglected Tropical Diseases (NTD) of the World Health Organisation (WHO). This study has been approved by the Ethics Committee of the Universitat Oberta de Catalunya.

Please take time to read the following information carefully and discuss it with others if you wish. Feel free to ask us if there is anything that is not clear, or if you would like to receive more information (you will find our contact details at the end of the text). Take time to decide whether or not you wish to participate.

*2. “What is the purpose of this study?”*

The aim of this study is to conduct a cross-sectional research study to assess the quality of SkinNTDs App version 3 developed by Universal Doctor for WHO in collaboration with NLR as a decision supporting tool for the frontline health workers (FHW) in order to correctly diagnose and manage sNTDs. This app is being developed with the purpose of aiding FHW (especially those located in resource poor settings) to come up with diagnoses when dealing with patients who suffer from Neglected Tropical Diseases that primarily affect the skin.

With this study we want to obtain information about how the app performs in the field, whether or not it is helpful to its users, if it needs changes, and what those changes could be. This information can only be obtained from the target users of the app, which is why your participation is precious to us.

The importance of this study resides in its capacity to provide feedback to the developers of the app, so that they can make better versions that would eventually allow it to achieve its goal of being a highly performant diagnosing tool.

*3. “Why have I been invited to participate in this study?”*

You are eligible to participate in this study because you have the profile required for using Skin NTDs app. As we previously mentioned, the target audience for this app are frontline health workers who deal with patients who suffer from Skin NTDs. In order to fill the survey (which is the first part of our study) you will be required to download the app and use it for at least one week (if the deadline allows it). Since you will be already familiar with the App that makes you the perfect candidate for our interview.

*4. “What if I don’t want to take part in this study, or if I want to withdraw later?”*

Participation in this study is voluntary. It is completely up to you whether or not you want to take part in it. You can decide to withdraw any time without having to give a reason. If you do so, we will automatically discard all the information you have given us.

*5. “How will my confidentiality be protected?”*

Following the “*Ley Orgánica de Protección de Datos 3/2018”* and the *Regulation (EU) 2016/679 of the European Parliament and of the Council of 27 April 2016 on the protection of natural persons with regard of the processing of personal data and on the free movement of such data*, any identifiable information that is collected about you in connection with this study will remain confidential, and will be disclosed only with your permission, or except as required by law. Only the researchers named above, and the Ethics Committee can have access to your details.

*6. “What does this study involve?”*

If you agree to join us in this study, you will be asked to answer an online survey and afterwards you will be invited to participate in a semi-structured interview, as mentioned above. The online survey consists of 45 closed questions that will not take you longer than 10 minutes. Afterwards the interview will consist of a short teleconference (with or without video, according to your preferences), where you will be asked to give your opinion on the SkinNTDs app, by answering a series of questions that we have prepared. In order to collect the information thoroughly, the interview will be recorded. However, once the data is processed, all the recordings obtained in this study will be deleted.

*7. “Is there any risk to take part in this study?”*

Since this study does not involve any intervention there are no risks or hazards to expect. However if during the study you feel any discomfort you have the right to withdraw from it as mentioned before or contact the principal investigator.

*8. “Will I benefit from the study?”*

With the information we receive from all the feedback we get from this study, the developers of theSkin NTDs app will be more aware of the needs of its users. That means that the app will be able to better serve its purpose; and ultimately, if you keep on using it, it could help you in your diagnosing task, when dealing with patients that are affected by Skin NTDs.

*9. “Will taking part in this study cost me anything, and will I be paid?”*

Participation in this study will not cost you anything, nor will it retribute you for taking part in it.

*10. “What happens with the results?”*

The results of this study will be published only for research issues. Your opinion and feedback will be completely anonymized.

If you wish, the results of the study can be provided to you.

*11. “Who should I contact if I have inquiries or if I have concerns about the conduct of this study?”*

This study has been approved by the Ethics Committee of the Universitat Oberta de Catalunya. For any concern you may have, you can contact them at comite_etica@uoc.edu.

If you wish to take part in this study, please give us your permission by filling the consent form you will find in the next page. Thank you for considering this study!

If you have any inquiries or if you want additional information, please contact Mireia Cano at [mireiacano94@gmail.com](mailto:mireiacano94@gmail.com)
