# Supplementary material for: Assessing the Quality of the World Health Organization’s Skin NTDs App as a Training Tool in Ghana and Kenya: Protocol for a Cross-sectional Study
Source: JMIR Res Protoc. 2022 Dec 8;11(12):e39393. doi: 10.2196/39393 (PMC9782345; doi:10.2196/39393)
Supplement: Multimedia Appendix 2 [file resprot_v11i12e39393_app2.docx]

**MULTIMEDIA APPENDIX 2. Semi-structured interview**

1. Brief presentation of the interviewer and the study
2. Which is your overall opinion of the *SkinNTDs* App?
3. Core questions:

- What features do you think are essential to the app? The features that you wouldn’t change in the app. Why?
- What features do you think are the least useful? The features you wouldn’t mind taking off the app. Why?
- What features must the app have necessarily in order for you to use it or recommend it to someone?
- Do you see the app being incorporated as a standard medical device in your workplace? Why?
- Do you use other healthcare apps in your daily practice? What about your colleagues?
- After using the App, who do you think is the ideal end-user?
- What do you think would be the best option to disseminate the App?
- Do you think that 5 days were enough time to test the App and be able to answer the MARS questionnaire?

1. Unplanned questions which arise based on participant’s answer.

- Would you like to add something else, or talk about another aspect that you feel is relevant to the app?
